# The Impact of HIV Viral Suppression and Immune Status on Rifampicin-Resistant Tuberculosis Outcomes: A Systematic Review and Meta-Analysis Protocol

**DOI:** 10.3390/tropicalmed11060160

**Published:** 2026-06-15

**Authors:** Tukisho Mphahlele, Thendo Gertie Makhado, Lufuno Makhado

**Affiliations:** 1 Department of Public Health, Faculty of Health Sciences, University of Venda, Thohoyandou 0950, South Africa; lufuno.makhado@univen.ac.za; 2 Department of Advanced Nursing Sciences, Faculty of Health Sciences, University of Venda, Thohoyandou 0950, South Africa; gertie.makhado@univen.ac.za

**Keywords:** rifampicin-resistant tuberculosis, HIV viral suppression, impact, immune status, treatment outcome, HIV co-infection

## Abstract

**Background/Objectives:** Rifampicin-resistant tuberculosis (RR-TB) and HIV co-infection remain major contributors to morbidity and mortality, particularly in high-burden settings. HIV-related clinical factors, including viral suppression, CD4-defined immune status, HIV drug resistance, virological failure, and ART failure, may influence RR-TB treatment response; however, existing evidence remains fragmented. This systematic review and meta-analysis protocol aims to synthesize evidence on the impact of HIV viral suppression, immune status, and HIV drug resistance/ART resistance status on RR-TB treatment outcomes. **Methods:** This protocol was developed in accordance with the Preferred Reporting Items for Systematic Reviews and Meta-Analyses Protocols guidelines. Published peer-reviewed studies and relevant grey literature from January 2005 to December 2025 will be searched in PubMed/MEDLINE, Cochrane Library, Embase, Web of Science, ScienceDirect, EBSCOhost, PsycINFO, Google Scholar, and other relevant sources. No language restriction will be applied at the search stage. Where feasible, non-English records will be translated for title/abstract and full-text screening. Two reviewers will independently screen studies, extract data, and assess study quality, with disagreements resolved by a third reviewer. Study-level risk of bias will be assessed using design-appropriate tools, and the certainty of evidence for each outcome will be evaluated using GRADE. **Results:** Evidence will be synthesized narratively and, where studies are sufficiently homogeneous, quantitatively through meta-analysis. Outcomes of interest will include treatment success, treatment failure, mortality, treatment completion, microbiological cure, and adverse events. Subgroup analyses will be considered by viral suppression status, CD4-defined immune status, HIV drug resistance/ART resistance status, geographic region, and treatment regimen where data permit. **Conclusions:** This review will provide evidence on how HIV viral suppression, immune status, and HIV drug resistance/ART resistance influence RR-TB treatment outcomes. The findings may inform integrated TB/HIV care, clinical monitoring, and treatment strategies for individuals co-infected with HIV and RR-TB.

## 1. Introduction

Tuberculosis (TB), caused by *Mycobacterium tuberculosis*, continues to be one of the leading causes of illness and death worldwide, particularly in low- and middle-income countries where healthcare resources are constrained [1,2]. The global fight against TB has been further undermined by the emergence and spread of drug-resistant forms, notably rifampicin-resistant TB (RR-TB) and multidrug-resistant TB (MDR-TB), which are resistant to both rifampicin and isoniazid [3]. According to the World Health Organization, drug-resistant tuberculosis remains a major global public health concern, with rifampicin-resistant and multidrug-resistant TB continuing to threaten progress toward global TB control targets [4].

These drug-resistant strains represent a serious threat to global TB control efforts, highlighting the urgent need for effective diagnostic, treatment, and prevention strategies.

The convergence of the HIV and TB epidemics, particularly in regions with high burdens of both infections, poses a profound challenge to global health systems. HIV infection significantly increases the risk of developing active TB following exposure to *M. tuberculosis* and worsens clinical outcomes once infection occurs. The co-existence of HIV and RR-TB creates an especially severe clinical and public health burden, as individuals co-infected with these diseases experience substantially higher rates of treatment failure, relapse, and mortality compared to those infected with TB alone [5,6]. This can be attributed largely to the immunosuppressive nature of HIV, which compromises host immune defences and promotes rapid TB disease progression.

While standard treatment regimens containing rifampicin are highly effective for drug-susceptible TB, their utility in cases of RR-TB is significantly limited. Patients with RR-TB must rely on second-line drugs that are often more toxic, less effective, and require longer treatment durations [7,8]. These factors contribute to poorer adherence, increased adverse events, and a heightened risk of further resistance. The combined burden of HIV-related immunosuppression, drug resistance, and treatment toxicity contributes to persistently high morbidity and mortality rates among co-infected populations, especially in resource-limited settings.

### Rationale and Significance

Despite the substantial global burden of RR-TB and HIV co-infection, important knowledge gaps remain regarding the influence of HIV-related clinical factors—such as viral suppression, immune status (CD4 count), and HIV drug resistance—on RR-TB treatment outcomes. Existing studies provide evidence that HIV-positive individuals with RR-TB experience worse treatment outcomes; however, these findings remain fragmented and lack a comprehensive synthesis across diverse settings and populations. Consequently, there is insufficient evidence to inform tailored clinical management and policy interventions for this vulnerable group [9,10]. Specifically, the complex interactions between HIV and *Mycobacterium tuberculosis* exacerbate disease progression and complicate treatment, often leading to higher viral loads and more pronounced immune activation in co-infected individuals [11]. This dual infection also accelerates the decline of immunological functions, leading to increased susceptibility to opportunistic infections and heightened mortality if left untreated [12].

A nuanced understanding of how HIV viral suppression and immune function affect RR-TB treatment response is essential for improving therapeutic outcomes and guiding integrated care approaches. Such evidence is particularly critical in settings with high HIV/TB co-burden, where treatment success is hindered by delayed diagnosis, limited drug availability, and challenges in adherence support [13]. This systematic review will therefore collate and critically appraise existing evidence to elucidate the relationship between HIV viral suppression, immune status, and RR-TB outcomes.

By systematically synthesizing data across studies, this review will contribute to the global evidence base necessary to refine clinical guidelines, strengthen treatment programs, and support the development of context-specific strategies for improving outcomes among people co-infected with HIV and RR-TB. Ultimately, this work aims to inform more effective, evidence-based policies and interventions that address the intersecting challenges of HIV and rifampicin-resistant tuberculosis, thereby reducing morbidity and mortality and enhancing quality of life for affected individuals [14,15].

## 2. Purpose

The purpose of this systematic review is to explore the impact of HIV viral suppression and immune status on rifampicin-resistant tuberculosis outcomes.

### Specific Objectives

- To determine the influence of CD4-defined immune status on rifampicin-resistant tuberculosis treatment outcomes among PLHIV.
- To assess the impact of HIV viral load and viral suppression status on rifampicin-resistant tuberculosis treatment outcomes.
- To examine the association between HIV drug resistance, ART resistance, virological failure, or ART failure and rifampicin-resistant tuberculosis treatment outcomes.
- To compare rifampicin-resistant tuberculosis treatment outcomes between HIV-suppressed and HIV-unsuppressed individuals across HIV drug resistance/ART resistance status and global regions.

## 3. Materials and Methods

This systematic review and meta-analysis will examine the impact of HIV viral suppression, CD4-defined immune status, HIV drug resistance, ART resistance, virological failure, and ART failure on treatment outcomes among individuals co-infected with HIV and rifampicin-resistant tuberculosis. The protocol follows the Preferred Reporting Items for Systematic Reviews and Meta-Analyses Protocols guidelines [16]. The completed PRISMA-ScR checklist is provided in Supplementary Table S1. The review will synthesize evidence from published peer-reviewed studies and relevant grey literature from January 2005 to December 2025. The review will include studies conducted globally, including both high-income and low- and middle-income countries.

### 3.1. Eligibility Criteria

#### 3.1.1. Review Question (PICO Framework)

The review question will be guided by the PICO (Population, Intervention, Comparison, and Outcomes) framework, as provided hereunder.

- P (Population): HIV-positive individuals co-infected with rifampicin-resistant TB (including pulmonary and extrapulmonary forms).
- I (Intervention): Antiretroviral therapy (ART), TB treatment regimens.
- C (Comparison): Outcomes in individuals with suppressed versus unsuppressed HIV viral load, with higher versus lower CD4-defined immune status, and with versus without documented HIV drug-resistance mutations, ART resistance, virological failure, or ART failure where reported.
- O (Outcomes): RR-TB treatment outcomes, including treatment success, microbiological cure, treatment completion, treatment failure, mortality, loss to follow-up, relapse where reported, and adverse events.

Therefore, the review question will be: In individuals co-infected with HIV and rifampicin-resistant tuberculosis, how do HIV viral load or viral suppression status, CD4-defined immune status, HIV drug resistance, ART resistance, virological failure, or ART failure influence RR-TB treatment outcomes, including treatment success, treatment failure, mortality, treatment completion, microbiological cure, and adverse events?

#### 3.1.2. Inclusion Criteria

Inclusion criteria aligning with the PICO framework are included in Table 1.

#### 3.1.3. Exclusion Criteria

Studies will be excluded if they do not meet the eligibility criteria, including studies that address HIV or rifampicin-resistant tuberculosis independently without reporting outcomes for co-infected individuals, studies that do not report RR-TB treatment outcomes, editorials, commentaries, opinion pieces, and studies published before January 2005. Records will not be excluded on the basis of language at the search stage. Where translation is not feasible after reasonable attempts, this will be documented and reported transparently in the review process.

## 4. Information Sources

A comprehensive literature search will be conducted across multiple electronic databases to ensure a wide and inclusive evidence base for this systematic review. The databases to be searched include PubMed/MEDLINE, Cochrane Library, EMBASE, Web of Science, ScienceDirect, EBSCOhost (CINAHL), PsycINFO, and Google Scholar.

In addition to electronic database searches, we will use hand searching to identify relevant studies not indexed in the selected databases. This will include manually screening the reference lists of included articles and targeted scanning of key journals focusing on HIV and tuberculosis. These supplementary techniques enhanced the sensitivity of our search strategy and helped ensure that pertinent peer-reviewed studies were not overlooked.

To manage and organize the retrieved references effectively, EndNote reference management software will be used. This software will automatically identify and remove duplicate records, ensuring accuracy and efficiency in the reference list. The de-duplicated dataset will then be exported into Covidence systematic review software for screening, data extraction, and synthesis. This dual-platform approach will ensure a rigorous and transparent review process.

## 5. Search Strategy

The search strategy will be developed using a combination of controlled vocabulary (e.g., MeSH terms) and free-text terms [16]. It will employ Boolean operators (AND, OR) to combine search terms across multiple concepts, ensuring comprehensive coverage of the literature relevant to HIV, rifampicin-resistant tuberculosis, and treatment outcomes.

The search strategy will be structured around six key thematic clusters of keywords as outlined below:

1. Rifampicin-resistant tuberculosis-related terms: “Rifampicin-resistant tuberculosis,” “RR-TB,” “rifampicin mono-resistant TB,” “RmR-TB,” “MDR/RR-TB.”
2. HIV infection-related terms: “HIV infection,” “human immunodeficiency virus,” “HIV disease,” “HIV-positive,” “Retroviral disease,” “AIDS.”
3. People living with HIV-related terms: “People living with HIV,” “PLWHIV,” “PLHIV,” “PLWHA,” “PLWH,” “PLWA.”
4. TB/HIV co-infection-related terms: “TB/HIV,” “HIV/TB,” “TB-HIV co-infection.”
5. Rifampicin-resistant TB treatment outcome-related terms: “Rifampicin-resistant treatment outcome,” “treatment success,” “treatment failure,” “mortality.”
6. HIV-related clinical and treatment terms: “immune suppression,” “immunosuppression,” “CD4 count,” “CD4+ T-cell count,” “viral load,” “viral suppression,” “virological suppression,” “virological failure,” “ART failure,” “antiretroviral therapy failure,” “HIV drug resistance,” “ART resistance,” “antiretroviral resistance,” “HIV resistance mutations,” and “HIV mutations.”

Boolean operators and truncation symbols will be used appropriately to expand or limit search results. The search will be limited to studies published between January 2005 and December 2025. No language restriction will be applied at the search stage. Non-English titles and abstracts will be screened using translation tools or available translation support where feasible. Full texts of potentially eligible non-English studies will be translated where possible, and studies that cannot be translated after reasonable attempts will be documented in the study selection process.

## 6. Study Selection

The study selection process will be conducted systematically and transparently to ensure methodological rigor. The process will begin by importing all retrieved studies into EndNote, and then removing duplicates. The deduplicated studies will then be exported to Covidence for further screening and management.

The initial screening will involve the primary reviewer (TM) reviewing titles and abstracts to identify potentially eligible studies. This will be followed by full-text screening of the shortlisted articles by both TM and the supervisor (TGM). Cohen’s kappa statistic will be used to assess inter-rater agreement among the reviewers and measure the degree of agreement beyond chance. Each reviewer will independently assess the studies for inclusion against the predefined eligibility criteria. In cases where disagreements arise over study inclusion, a third reviewer (LM) will be consulted to reach a consensus. This three-tiered review approach ensures objectivity, minimizes bias, and strengthens the reliability of study selection.

The entire selection process will be documented using a PRISMA flow diagram, which will illustrate the number of records identified, screened, excluded (with reasons), and ultimately included in the review [16]. This will ensure transparency and reproducibility of the study selection process.

## 7. Data Charting Process

Data will be extracted using the Covidence systematic review management tool. According to Li, Higgins [17], the extraction process will follow five structured steps: planning, piloting, extracting, comparing, and reaching consensus before exporting. This process ensures systematic and transparent data handling.

### 7.1. Planning

The planning stage involves designing the data extraction form and determining the key information to collect (e.g., study design, population, interventions, and outcomes). The PICOS framework will guide the development of the extraction form to ensure that all variables relevant to rifampicin-resistant tuberculosis (RR-TB) and HIV co-infection are captured.

The data extraction template will include details of studies focusing on RR-TB and HIV co-infection, comparisons of HIV-related factors (viral suppression, immune status, resistance mutations), and RR-TB treatment outcomes. This structured plan ensures consistency among reviewers and guarantees that extracted data align with the review’s PICOS-based objectives. Data extraction will strictly follow the defined inclusion and exclusion criteria.

The extraction form will also capture HIV-related clinical and treatment variables, including ART regimen, ART duration where reported, HIV viral load, viral suppression threshold used by each study, CD4 count or CD4 category, HIV drug-resistance mutations, ART resistance, virological failure, ART failure, timing of ART in relation to RR-TB treatment, and drug–drug interaction considerations where reported. Study language, translation method, and whether translation was required for screening or extraction will also be documented.

- Software: Covidence will serve as the primary extraction platform.
- Predefined Variables: These are system variables already embedded in Covidence.
- Population characteristics: all genders, all age groups, global settings.
- Publication status: published studies.
- Language: No language restriction at the search stage; language of publication and translation method will be recorded.
- Date range: January 2005–December 2025.

### 7.2. Piloting

The extraction form will be tested on a subset of eligible studies to ensure clarity, relevance, and functionality. This pilot process will assess whether the data collection tool effectively captures all necessary information aligned with the review question. Adjustments will be made as needed to enhance data quality and relevance.

### 7.3. Extraction

The finalized form will then be used for systematic extraction of relevant data from all included studies. Both reviewers (TM and TGM) will perform dual-independent extraction to enhance accuracy and reduce bias.

Data will be captured for both quantitative and qualitative variables, including study characteristics, population characteristics, RR-TB diagnostic criteria, RR-TB treatment regimen, ART regimen, HIV viral load, viral suppression threshold, CD4 count or CD4 category, HIV drug-resistance mutations, ART resistance, virological failure, ART failure, timing of ART initiation, and RR-TB treatment outcomes. The comment field within Covidence will be used to annotate inclusion/exclusion decisions and observations for specific studies.

### 7.4. Comparison, Consensus, and Export

Following the extraction, the reviewers will compare entries to identify discrepancies. Disagreements will be resolved through discussion; if consensus cannot be reached, a third reviewer (LM) will adjudicate.

Once finalized, the dataset will be exported from Covidence into Excel for synthesis and subsequent statistical analysis. This approach ensures consistency, transparency, and reproducibility of the dataset used in both narrative and quantitative synthesis.

## 8. Quality Assessment

Methodological quality and risk of bias will be assessed at the study level using tools appropriate to each included study design. Randomized controlled trials will be assessed using the Cochrane Risk of Bias 2 tool, non-randomized intervention studies using ROBINS-I, and observational cohort or case–control studies using an appropriate validated observational-study appraisal tool, such as the Newcastle–Ottawa Scale or the Joanna Briggs Institute critical appraisal tools. The selected tool for each study will be documented and justified in accordance with the study design.

Two reviewers will independently assess risk of bias, with disagreements resolved through discussion or consultation with a third reviewer. Domains assessed will include selection bias, measurement of exposure and outcomes, confounding, missing data, selective reporting, and other design-specific sources of bias.

The certainty of evidence for each main outcome will then be assessed using the GRADE approach. GRADE will be applied at the outcome level rather than as a study-level appraisal tool. Certainty will be rated as high, moderate, low, or very low based on risk of bias, inconsistency, indirectness, imprecision, and publication bias. A GRADE Summary of Findings table will be prepared for the main outcomes, including treatment success, treatment failure, mortality, treatment completion, microbiological cure, and adverse events.

## 9. Data Synthesis

### 9.1. Narrative Synthesis

A narrative synthesis will be conducted for studies that are clinically, methodologically, or statistically heterogeneous and therefore unsuitable for quantitative pooling. The synthesis will summarise study characteristics, populations, HIV-related clinical factors, RR-TB treatment regimens, and reported outcomes in relation to the review objectives [18].

This method allows for a comprehensive and nuanced presentation of each study’s findings, particularly focusing on the complex interplay between rifampicin-resistant tuberculosis (RR-TB) and HIV co-infection.

The narrative synthesis will focus on how CD4-defined immune status, HIV viral load or viral suppression status, HIV drug resistance, ART resistance, virological failure, and ART failure are associated with RR-TB outcomes. Findings will be grouped by key outcomes, including treatment success, treatment failure, mortality, treatment completion, microbiological cure, and adverse events.

The interpretation of narrative findings will consider study design, risk of bias, population characteristics, outcome definitions, and consistency of findings across settings. This approach will allow the review to identify patterns in the evidence while acknowledging differences in study methods and clinical contexts.

The narrative synthesis will serve as a foundational step, preceding any statistical analysis. This initial storytelling approach will clarify the anticipated results and illuminate the underlying themes and patterns that emerge from the literature. By doing so, we aim to enhance our understanding of how these studies inform the subsequent meta-analysis, ensuring that the synthesis not only reflects the complexity of the relationships between RR-TB and HIV co-infection but also guides the interpretation of subsequent quantitative analyses. Overall, this comprehensive methodology aims to contribute meaningfully to the discourse on better management strategies for individuals affected by RR-TB and HIV co-infection.

### 9.2. Meta-Analysis

Where studies are sufficiently homogeneous in terms of population, exposure definitions, comparator groups, and outcomes, quantitative synthesis will be conducted using meta-analysis [19]. Studies will be grouped according to the HIV-related exposure of interest, including viral suppression status, CD4-defined immune status, HIV drug resistance/ART resistance status, virological failure, and ART failure.

The studies will be systematically categorized into two primary groups: those that can be directly compared and those that indicate relationships among variables. Depending on the outcome measures reported across studies, effect sizes will be presented as pooled odds ratios (ORs), risk ratios (RRs), or hazard ratios (HRs) with 95% confidence intervals.

To evaluate the effect size of each study, a random-effects model will be used. This model is particularly valuable as it accounts for variability both within and between studies, providing a more generalized estimate of effects. Additionally, Cochran’s Q test and the I2 statistic will be used to evaluate statistical heterogeneity among studies to determine whether changes in research findings are due to random error or to real variations in effect sizes. Funnel plots and Egger’s regression test will be used to assess publication bias when sufficient studies are available. Where data permit, subgroup analyses will be conducted by HIV viral suppression status, CD4 category, HIV drug resistance/ART resistance status, ART regimen, RR-TB treatment regimen, age group, geographic region, and study design. Sensitivity analyses will be used to assess the influence of studies at high risk of bias, studies requiring translation, and studies using different definitions of viral suppression or treatment outcomes.

Furthermore, a sensitivity analysis will be conducted to assess the robustness of the findings. This analysis will evaluate how the inclusion or exclusion of specific studies affects the overall results, with particular focus on those that may be contentious or have led to disagreements among researchers. By examining the influence of these studies on the overall conclusions, researchers can better understand the stability of the findings and ensure that the meta-analysis provides reliable and valid insights.

This structured approach to meta-analysis will enhance the clarity of the synthesized findings and contribute significantly to the advancement of knowledge in the field by providing a thorough and critical evaluation of the studies in question.

## 10. Planned Presentation of the Findings

The presentation of results for this systematic review and meta-analysis will adhere to the PRISMA 2020 guidelines [16], ensuring transparency and reproducibility. The overall selection process, from identification through screening and inclusion, will be depicted using a PRISMA flow diagram, which will clearly illustrate the number of records retrieved, duplicates removed, articles screened and excluded (with reasons), and those finally included for qualitative and quantitative synthesis.

Results will be presented in both narrative and statistical formats. The narrative synthesis will provide a detailed summary of key study characteristics, methodological quality, and the thematic findings relating to the impact of HIV viral suppression and immune status (CD4 count) on rifampicin-resistant tuberculosis (RR-TB) outcomes. The findings will be organized around the main review objectives, with an emphasis on treatment success, failure, mortality, and adverse events among co-infected individuals.

Quantitative results from the meta-analysis will be displayed using forest plots, summarizing effect sizes such as pooled odds ratios (ORs) or relative risks (RRs) with 95% confidence intervals (CIs). Funnel plots will be generated to visually assess potential publication bias, supplemented by Egger’s regression test for quantitative evaluation. Where possible, subgroup analyses will be presented to explore differences based on HIV viral suppression status, CD4-defined immune status, HIV drug resistance/ART resistance status, virological failure, ART failure, ART regimen type, RR-TB regimen, study design, and geographic region.

The quality and certainty of evidence will be summarized using a GRADE “Summary of Findings” table, which will provide a concise overview of the strength of evidence and confidence in effect estimates for each outcome [20]. Each table will include judgments on risk of bias, consistency, precision, and directness.

Collectively, the results will be synthesized to provide an integrated understanding of how HIV-related clinical parameters influence RR-TB treatment outcomes, with attention to contextual variations across geographic regions and study designs. The results of this review will be published in peer-reviewed journals and will form part of a postgraduate dissertation submitted to the University of Venda library database. The final report will contribute to global evidence on dual HIV/TB management strategies and support evidence-based interventions aimed at improving clinical outcomes in co-infected populations.

This systematic review and meta-analysis protocol has been registered with PROSPERO under registration number CRD420251148230, confirming compliance with international standards for systematic review registration and reporting.

## 11. Conclusions

The intersection of HIV infection and rifampicin-resistant tuberculosis (RR-TB) remains one of the most critical global health challenges, particularly in sub-Saharan Africa and other high-burden regions. Despite substantial progress in expanding antiretroviral therapy (ART) coverage and implementing directly observed treatment, the dual epidemics of HIV and RR-TB continue to contribute significantly to morbidity and mortality worldwide.

This systematic review and meta-analysis is expected to provide critical insights into the influence of HIV viral suppression, CD4-defined immune status, HIV drug resistance, ART resistance, virological failure, and ART failure on RR-TB treatment outcomes [21,22]. By synthesizing and, where appropriate, pooling available evidence, the review will address a key knowledge gap and support more integrated clinical approaches for people co-infected with HIV and RR-TB.

One of the key anticipated contributions of this review is to delineate how HIV viral suppression achieved through ART influences treatment success rates for RR-TB. Studies have suggested that achieving and maintaining viral suppression significantly reduces all-cause mortality among co-infected patients, likely through partial restoration of immune function and improved tolerance to anti-TB drugs [23]. Conversely, unsuppressed viral loads and persistent immune suppression (low CD4 counts) have been associated with delayed sputum conversion, prolonged infectiousness, and poor treatment adherence [24]. Quantifying these associations through meta-analysis will help determine the magnitude of such effects, providing evidence-based thresholds for monitoring HIV and TB treatment concurrently.

From a public health policy perspective, understanding the combined impact of HIV viral suppression and immune reconstitution on RR-TB outcomes is essential for guiding the WHO End TB Strategy and UNAIDS 95–95–95 goals. Integrating routine HIV viral load monitoring and CD4 assessments into RR-TB management protocols could enhance individualized treatment planning and improve survival rates [25]. This review will thus contribute to the optimization of clinical management frameworks by supporting differentiated care models for RR-TB/HIV co-infected populations.

Furthermore, this review’s findings may inform clinical decision-making in the context of drug–drug interactions between rifampicin-based TB regimens and ART. Such interactions are known to reduce plasma concentrations of several antiretroviral agents, thereby compromising viral suppression [15]. By summarizing evidence on these pharmacological relationships, this study can support revisions to national treatment guidelines and the development of safer combination regimens that minimize adverse interactions.

Another anticipated contribution lies in the methodological domain. By applying GRADE for evidence appraisal and PRISMA 2020 for transparent reporting [16,20], this review upholds rigorous international standards, ensuring the credibility and reproducibility of its findings. The combination of narrative synthesis for heterogeneous data and meta-analysis for comparable outcomes will allow both breadth and depth of interpretation, strengthening the translational relevance of the evidence base.

Ultimately, the results of this systematic review are expected to bridge the gap between research and practice by generating actionable evidence that supports early detection, immune monitoring, and integrated therapy for individuals co-infected with HIV and RR-TB. This aligns with current global health priorities advocating for person-centered, evidence-informed care to curb the dual epidemic’s impact [26,27]. By consolidating diverse findings into a unified framework, this review will help inform national TB/HIV programs, shape future clinical trials, and support global efforts toward the elimination of TB and HIV-related mortality by 2030.

## Figures and Tables

**Table 1:** Inclusion criteria for study selection.

PICO Element	Description of Criteria
i. Population group	Population groups infected with both HIV and rifampicin-resistant tuberculosis, including pulmonary and extrapulmonary RR-TB, across all age groups, genders, and geographic regions. Studies published from January 2005 to December 2025 will be considered.
ii. Interventions (treatment regimens)	ART treatment and RR-TB treatment regimens.
iii. Comparison group	Individuals with suppressed versus unsuppressed HIV viral load, with higher versus lower CD4-defined immune status, and with versus without documented HIV drug-resistance mutations, ART resistance, virological failure, or ART failure where reported.
iv. Outcome of interest	Rifampicin-resistant tuberculosis treatment outcomes, including treatment success, microbiological cure, treatment completion, treatment failure, mortality, loss to follow-up, relapse where reported, and adverse events.

## Data Availability

No new data will be generated.

## Supplementary Materials

The following supporting information can be downloaded at: https://www.mdpi.com/article/10.3390/tropicalmed11060160/s1. Table S1: Preferred Reporting Items for Systematic reviews and Meta-Analyses extension for Scoping Reviews (PRISMA-ScR) Checklist. Reference [28] is cited in the Supplementary Materials.

## Abbreviations

AIDS	Acquired Immune Deficiency Syndrome
ART	Antiretroviral Therapy
ARV	Antiretroviral (drug)
CD4	Cluster of Differentiation 4 (T-lymphocyte count)
CI	Confidence Interval
DOTS	Directly Observed Therapy, Short-course
GRADE	Grading of Recommendations Assessment, Development, and Evaluation
HIV	Human Immunodeficiency Virus
I2	I-squared statistic (measure of heterogeneity)
LM	Third Reviewer/Independent Adjudicator
MDR-TB	Multidrug-Resistant Tuberculosis
OR	Odds Ratio
PICO	Population, Intervention, Comparison, and Outcomes framework
PICOS	Population, Intervention, Comparison, Outcomes, and Study Design framework
PLHIV	People Living with HIV
PLWHA	People Living with HIV/AIDS
PRISMA	Preferred Reporting Items for Systematic Reviews and Meta-Analyses
PRISMA-P	Preferred Reporting Items for Systematic Reviews and Meta-Analyses Protocols
PROSPERO	International Prospective Register of Systematic Reviews
RCT	Randomized Controlled Trial
RR	Relative Risk
RR-TB	Rifampicin-Resistant Tuberculosis
TB	Tuberculosis
TGM	Supervisor/Second Reviewer
TM	Primary Researcher/First Reviewer
WHO	World Health Organization
